# Strain-dependent profile of misfolded prion protein aggregates

**DOI:** 10.1038/srep20526

**Published:** 2016-02-15

**Authors:** Rodrigo Morales, Ping Ping Hu, Claudia Duran-Aniotz, Fabio Moda, Rodrigo Diaz-Espinoza, Baian Chen, Javiera Bravo-Alegria, Natallia Makarava, Ilia V. Baskakov, Claudio Soto

**Affiliations:** 1 Mitchell Center for Alzheimer’s disease and Related Brain Disorders, Dept. of Neurology, University of Texas Houston Medical School, Houston, TX 77030, USA; 2 Innovative Drug Research Centre, Chongqing University, Chongqing 401331, China; 3 Universidad de los Andes, Facultad de Medicina, Av. San Carlos de Apoquindo 2200, Las Condes, Santiago, Chile; 4 Department of Laboratory Animal Science, School of Basic Medical Science, Capital Medical University, Beijing, 100069, China; 5 BioMET and Department of Anatomy and Neurobiology, University of Maryland School of Medicine, Baltimore, Maryland, USA

## Abstract

Prions are composed of the misfolded prion protein (PrP^Sc^) organized in a variety of aggregates. An important question in the prion field has been to determine the identity of functional PrP^Sc^ aggregates. In this study, we used equilibrium sedimentation in sucrose density gradients to separate PrP^Sc^ aggregates from three hamster prion strains (Hyper, Drowsy, SSLOW) subjected to minimal manipulations. We show that PrP^Sc^ aggregates distribute in a wide range of arrangements and the relative proportion of each species depends on the prion strain. We observed a direct correlation between the density of the predominant PrP^Sc^ aggregates and the incubation periods for the strains studied. The relative presence of PrP^Sc^ in fractions of different sucrose densities was indicative of the protein deposits present in the brain as analyzed by histology. Interestingly, no association was found between sensitivity to proteolytic degradation and aggregation profiles. Therefore, the organization of PrP molecules in terms of the density of aggregates generated may determine some of the particular strain properties, whereas others are independent from it. Our findings may contribute to understand the mechanisms of strain variation and the role of PrP^Sc^ aggregates in prion-induced neurodegeneration.

PrP^Sc^, the disease associated isoform of prions, is a polymer composed of many units of this protein arranged in an inter-molecular β-sheet conformation^1^,^2^. The accumulation of these particles in the brain is linked to neuronal death, synaptic alterations, brain inflammation and spongiform degenerationleading to various forms of prion diseases in humans and animals^1^,^3^. Despite its low incidence in humans, animal prionopathies can reach larger numbers in species such as deer, sheep and cattle^4^,^5^,^6^. The zoonotic potential of some animal prion diseases represents a persistent threat for public health^7^.

The mechanisms of prion virulence have been partially described^8^,^9^. The most important process related to prion infectivity involves the recruitment of the cellular prion (PrP^C^), a constitutively expressed protein, into the growing PrP^Sc^ aggregate^1^,^10^. Misfolded prion polymers are thought to fragment, generating new “seeds” able to recruit monomeric PrP^C^ units and resulting in an exponential generation of the infectious agent^2^. As many natural and synthetic polymers, prion aggregates are thought to exist as a continuum of sizes, ranging from small oligomers to large fibrillar structures^11^,^12^,^13^,^14^. The profile of aggregation for prion polymers seems to depend on the specific conformation adopted by the constituent monomers^12^,^15^.

Different biochemical and biological properties have been associated with misfolded protein aggregates of different sizes. Amyloid plaques (mostly composed of to fibrillar structures) were the first pathological entities described in other Protein Misfolding Disorders (PMDs)^16^. For a long time, the presence of amyloid deposits has been used as the definitive (*post-mortem*) diagnosis for several PMDs. These fibrillar/dense aggregates were associated with cell toxicity, insolubility and resistance to biological clearance^16^. However, several forms of prion diseases in humans or experimental animals lack noticeable fibrillar PrP deposits as analyzed by histological or biochemical techniques^17^,^18^. Although soluble and more sensitive to degradation than their fibrillar counterparts, misfolded oligomers have been placed as the main culprits for the toxicity observed in several PMDs^19^,^20^. In fact, neurodegeneration in the brain of AD patients has been suggested to better correlate with oligomeric amyloid-β (Aβ) than amyloid plaques^21^. Small Aβ aggregates such as dimers, trimers and the so called Aβ*56 have been described as highly toxic *in vitro* and *in situ*^19^,^22^,^23^,^24^,^25^. In prion diseases, oligomers have also been identified as highly toxic^20^,^26^,^27^, as well as the most successful infectious entities^11^,^12^. The minimum prion infectious particle has been described to be as small as a dimer/pentamer, whereas the most infectious is formed by 14–28 monomeric units^11^. Fibrils are thought to act as reservoirs of these small pathological structures and as the natural end-point of the progressive growing of the aggregates^28^.

In a similar manner to conventional infectious agents, prions exhibit a large strain variation, resulting in diseases with distinct clinical symptoms, incubation periods, profile of neuropathological damage and biochemical properties of the agent^29^. Compelling evidence suggests that strain variation lies in the differential conformation that misfolded prions acquire, a property that is independent of the primary structure of the protein^29^. PrP^Sc^ associated with different strains has been shown to form distinct types of aggregates, a feature that could be connected to some of the peculiar characteristics observed for each agent^12^,^15^. In this sense, the presence of protease sensitive/low molecular weight structures has been linked to shorter incubation periods^12^. In the same line, it has been suggested that protease sensitive structures are indicators of a faster disease progression^30^,^31^. The bulky presence of amyloid deposits in asymptomatic GPI-less PrP mice further suggests that large structures are less toxic than small oligomers^32^. However, other reports have proposed that the relationship between size and pathological effects is not always balanced towards small oligomers, and that toxicity could be dependent not only on the size, but also in the particular conformational arrangement displayed by the aggregate^33^,^34^. The physicochemical pattern of PrP aggregates in disease has been only partially studied^15^ and their contribution to disease is still obscure. Although several protocols have been utilized to investigate the distribution profile of prion aggregates, many of them involve pre-treatments that may change the natural dynamic distribution of the particles^11^,^35^. Indeed, we have recently shown that proteolytic digestion or differences in salt concentration can substantially alter PrP properties, including the distribution of aggregates^36^. In this study we analyzed the aggregation profile, under conditions of minimal manipulation, of three hamster prion strains showing different clinical, histopathological and biochemical features.

## Methods

### Ethics Statement

All animal manipulations were performed according to NIH guidelines for animal research and approved by the Animal Welfare Committee of the University of Texas Medical School at Houston (HSC-AWC-08-161 and HSC-AWC-11-134) and the Institutional Animal Care and Use Committee of the University of Maryland, Baltimore (Assurance Number A32000-01; Permit Number 0309001).

### Animals, inocula preparation and infectivity studies

For this study we used Syrian Golden hamsters infected with either HY (Hyper-TME), DY (Drowsy-TME)^37^ or SSLOW (Synthetic Strain Leading to Overweight^38^,^39^,^40^) prion strains. Brain homogenates from terminally ill animals (stage 4 of clinical signs as explained below) were prepared at 10% w/v in Phosphate Buffer Saline (PBS) plus a cocktail of protease inhibitors using a glass manual homogenizer. Homogenates were spun down for 45 s at 805 × g to remove tissue debris. Resulting materials were used for fractionation studies or subsequent bioassays. For all prion infectivity assays, 40 days old female hamsters were intra-cerebrally (i.c.) inoculated under isofluorane anesthesia with infected brain homogenates and checked for clinical signs five times a week. Clinical signs were different for each strain and were measured using a scoring system as follows. HY: 1, normal animal; 2, mild behavioral abnormalities including hyperactivity and hypersensitivity to noise; 3, moderate behavioral problems including head tremors, ataxia, wobbling gait, head bobbing, irritability, and aggressiveness; 4, severe behavioral abnormalities including all of the above plus head and body jerks and spontaneous backrolls; and 5, terminal stage of the disease in which the animal lies in the cage and is no longer able to stand up. DY: 1, normal animal; 2, mild behavioral abnormalities including mild lethargy; 3, increased lethargy and curl up sleep posture; 4, same as 3 but decrease in body weight and difficulty to open the eyes; and 5, terminal stage of the disease in which the animal lies in the cage and is no longer able to stand up. SSLOW: 1, normal animal; 2, mild behavioral abnormalities including slight sensitivity to noise and touch; 3, same as 2 but increase in body weight and apparent skin dryness; 4, progressive increase in body weight, dry skin (sometimes including matted hair, especially in the thoracic area), and mild lethargy; and 5, terminal stage of the disease in which the animal have reduced body weight, lies in the cage and is no longer able to stand up. Animals scoring stage 4 for more than one week in HY and DY, and longer than 3 weeks in SSLOW, were sacrificed by CO_2_ inhalation, followed by decapitation. For HY prions, 40 days old female hamsters were injected as described above, sacrificed either at 60, 70 and 81 days post-infection (dpi) and resulting brain homogenates used for sucrose gradient fractionation studies. For all animals, right brain hemispheres were frozen at −80 °C (and stored until homogenates were prepared) and left hemispheres were fixed in Carnoy fixative for further histological studies.

### Sucrose gradient fractionation

150 μL of 10% w/v homogenates from uninfected (n = 3), HY (n = 5) and DY (n = 5) brains, or 5% w/v homogenates from SSLOW (n = 3) brains were mixed with 150 μL of 10% w/v sarkosyl solution (prepared in ultrapure water) and gently mixed for 5 minutes at room temperature (n numbers represent brain homogenates obtained from different animals). Samples were placed over a sucrose gradient prepared by adding 600 μL of 70%, 65%, 60%, 55%, 50%, 45% and 40% sucrose solutions from bottom to top in SW55Ti rotor tubes (Beckman, Brea, CA, USA). Sucrose solutions were prepared in TNS buffer (10 mM Tris, 150 mM NaCl, 1% Sarkosyl). Preparations were centrifuged at 202,300 × g for 24 h at 4 °C. Fractions were collected by carefully taking 300 μL from top to bottom. Pellets were resuspended in 300 μL of PBS by pipetting. All resulting fractions were mixed thoroughly and stored at −20 °C until use. 19 μL aliquots of each prion-derived fraction were either treated with Proteinase K (PK, 50 μg/mL final concentration) for 1 h at 37 °C with agitation, or kept untreated. PrP in each case was visualized by Western blot as explained below. PrP signals were measured using the Quantity One (4.6.7) software (BioRad, Hercules, CA, USA). Relative PrP signal in each fraction was expressed as a percentage of the total signal obtained for each fractionation assay. Results were plotted using the Prism GraphPad software (GraphPad, La Jolla, CA, USA).

### Histological procedures

Carnoy-fixed samples were dehydrated and included in paraffin. 10 μm tissue slices were mounted on glass slides, de-waxed with xylene and hydrated with solutions of decreasing strengths of alcohol (100% to 70%). For PrP^res^ (PK resistant PrP) staining, slides were rinsed with distilled water and treated with 10 μg/mL PK for 5 min. Samples were subsequently treated with 6% hydrogen peroxide (H_2_O_2_) for 20 min, rinsed in water, and finally subjected to 3 M guanidinium hydrochloride treatment (prepared in TrisHCl 10 mM, pH 7.8) for 20 min. Slides were incubated overnight using the 6H4 antibody (Prionics, Zurich, Switzerland) and non-specific binding prevented using Dako ARK (Dako, Glostrup, Denmark), following manufacturer’s recommendations. Immunostaining was developed using HRP-conjugated streptavidin visualized with DAB as chromogen. Tissue was later counterstained with haematoxylin for 30 s and rinsed in tap water for 10 min. For Thioflavin S staining, slides were treated with Thioflavin S (1% in ethanol 80%) for 10 min. Samples were washed in distilled water and rinsed quickly (20–30 dips) in 80% alcohol solution. Later, slides were dehydrated in ≥95% ethanol, cleared with xylene, and mounted with resinous mounting medium. Sections were examined under a bright field/epifluorescent DMI6000B Leica microscope (Leica, Buffalo Grove, IL, USA).

### PK resistance assay

10% brain homogenates were prepared as explained above and treated with the following PK concentrations: 50, 250, 500, 1250 and 2500 μg/mL. Samples were vortexed and incubated for 3 h at 37 °C with shaking (450 rpm) in a thermomixer (Eppendorf, Hauppauge, NY, USA). PK was inactivated by adding 50% volume of denaturing LDS (4X) buffer and heating at 95 °C for 10 min. PrP^27^,^28^,^29^,^30^ (disease-associated PK resistant PrP fragment) signal was visualized by Western blot as explained below and densitometry was calculated in relation to the lowest PK concentration (100%) using the Quantity One (4.6.7) software (BioRad, Hercules, CA, USA). Results were plotted using the Prism GraphPad software (GraphPad, La Jolla, CA, USA).

### Assessment of PK activity at different sucrose concentrations

A 10% w/v HY brain homogenate was mixed with a 10% w/v sarkosyl solution supplemented with protease inhibitors cocktail and centrifuged at 805 × g for 45 seconds at 4 °C. Pellet was discarded and supernatant was placed over a 20% sucrose cushion in a 5/6 ratio. Sample was centrifuged at 149,008 × g for 3 hours at 4 °C. Supernatant was discarded and the pellet was further resuspended in 0.1% Z-3,14 (prepared in PBS plus protease inhibitors). The resulting mix was placed over a 20% sucrose cushion and centrifuged again at 149,008 × g for 3 hours and 4 °C. Supernatant was discarded and pellet was submitted to the same resuspension/centrifugation step as previously explained. Resulting pellet was solubilized in deionized water (pH 8.5, supplemented with protease inhibitors), and placed over a 20% sucrose solution. This preparation was centrifuged at 149,008 × g for 3 hours at 4 °C. Pellet was finally resuspended by pipetting and sonication in a low volume of 20 mM Tris/HCl containing 1% sarkosyl at pH 8.5 (supplemented with protease inhibitors). Resulting material was highly concentrated and enriched in PrP^Sc^. 9 μL of fractions 3, 5, 8 and 14 obtained after centrifuging an uninfected hamster brain homogenate were mixed with 0.5 μL of the PrP preparation and 0.5 μL of PK (final concentration: 50 μg/mL). Samples were digested for 1 h at 37 °C with shaking. Reaction was stopped as explained above and the presence of PrP was visualized by Western blotting. Final signals were normalized in regards of the signal obtained after PK digesting the sample in PBS at the same conditions.

### Western blotting

Western blotting of PrP was performed as previously described^41^. Briefly, PK treated and un-treated prion fractions or brain homogenates, and spiked NBH fractions were mixed with 50% volume of denaturing LDS (4X) loading buffer (Invitrogen, Waltham, MA, USA). Samples were heated for 10 minutes at 95 °C and fractionated in 4–12% NuPAGE gels (Invitrogen, Waltham, MA, USA). Proteins were transferred to nitrocellulose membranes (GE Healthcare, Little Chalfont, UK) and probed with the 6D11 monoclonal antibody (Covance, Princeton, NJ, USA). After incubation with secondary antibody (GE Healthcare, Little Chalfont, UK) and washing, PrP was visualized by chemoluminescence using ECL plus (GE Healthcare, Little Chalfont, UK) in a dark chamber (BioRad, Hercules, CA, USA). For Western blot analyses of whole brain extracts, 1.5 mg equivalent of tissue per sample were analyzed.

### Statistical analyses

Data was expressed as means ± standard errors of the means from values obtained by a single fractionation procedure independently assessed per each brain homogenate. Descriptive statistics (Skewness and Kurtosis) were used to evaluate the distribution of PrP aggregates along the different fractions. Inferential statistical analyses were performed by Two-way ANOVA followed by Bonferroni post-tests. Statistical analyses were made using the GraphPad Prism 5.0 software. Statistical differences were considered significant for values of *p* < 0.05.

## Results

### Prion strains have different profiles of aggregation as studied by sucrose density gradients

**Figure 1:**
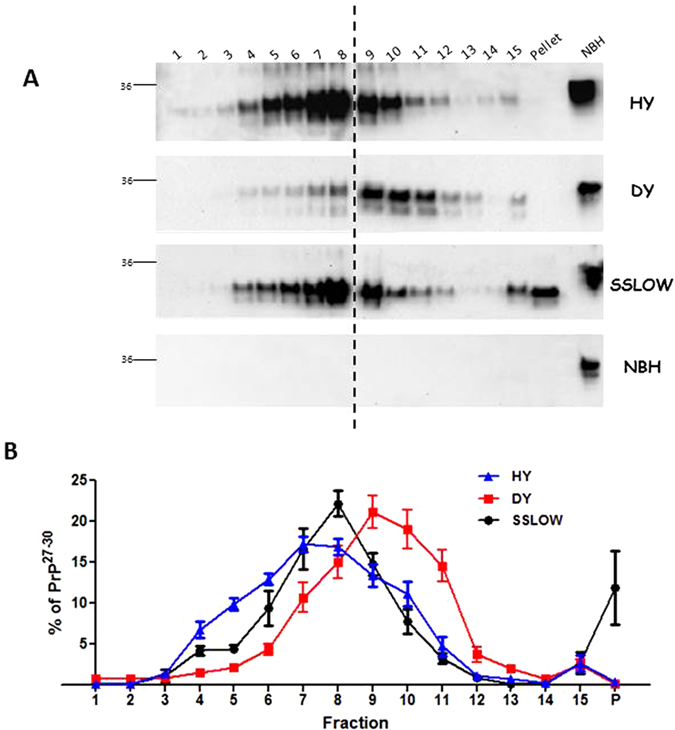
Sucrose-gradient fractionation of HY, DY and SSLOW prions. Brain homogenates from clinically sick (stage 4) hamsters were submitted to sucrose gradient fractionations coupled to ultracentrifugation. Fractionated samples were PK treated and analyzed by Western blot. (**A**) Representative blots from five (HY, DY) or three (SSLOW, NBH) fractionated brain extracts (obtained from different animals). Horizontal lines at the left of each blot represent a 36 KDa molecular weight marker. Vertical dotted lines depict splicing from different blots developed in the same membrane. Numbers at the top of the blots represent fraction numbers (from top to bottom). NBH: Normal (non-infected) brain homogenate. (**B**) Fractionation profiles for each prion strain as indicated. Values correspond to the average ± standard error calculated from values obtained by a single fractionation procedure per infected brain homogenates (n = 5 for HY and DY; n = 3 for SSLOW).

In order to test whether HY, DY, and SSLOW prions, having the same amino acid sequence, could be differentiated by their aggregate distribution profiles, we submitted samples from these three strains to 40–70% sucrose density gradients. Resulting preparations were ultracentrifuged for 24 hours and fractionated in 15 fractions plus the pellet. Each fraction was treated with PK and the PrP^27^,^28^,^29^,^30^ content was analyzed by Western blotting. Since any manipulation of the material may alter PrP properties, including the distribution of aggregates, pre-treatments were avoided as much as possible and limited only to a brief centrifugation to remove debris and addition of a mild detergent to break membranes and release molecules non-specifically bound to PrP aggregates (see Materials and Methods). We observed that aggregate distribution of PrP^27^,^28^,^29^,^30^ under these experimental conditions reaches equilibrium between 15 and 24 hours (tested up to 48 hours, data not shown). Although all three strains displayed a Gaussian-like distribution of protein aggregates, their profiles were easily differentiated (Fig. 1). DY prions comprised a population of denser aggregates (peak on fractions 9 and 10) when compared to HY prions (peaking at fractions 7 and 8). Although SSLOW prions had a similar distribution than the one observed for HY, this strain contained a strong signal in the pellet. As depicted in Supplementary Table 1, comparison of the percentage of PrP^27^,^28^,^29^,^30^ present in different fractions shows that each strain had a particular preference for certain arrangements. Whereas HY prions have the highest proportion of less dense aggregates (fractions 7–8), DY prions have a predilection for intermediate structures (9–10). Although similar in distribution compared to HY prions in the peak area, SSLOW prions exhibit the largest amount of material pelleting during centrifugation. Table 1 summarizes these differences by showing the preference of these strains for low- (fractions 4–5), intermediate- (fractions 10–11) or high- (pellet) density aggregates. Differences on the distribution of PrP^27^,^28^,^29^,^30^ along the fractions (skewness and kurtosis) for the three prion strains analyzed were found. The differential distribution of aggregates along the entire gradient for each particular prion strain was confirmed by two way ANOVA followed by Bonferroni post-test to analyze specific differences (Supplementary Table 2). When expressing the fractionation results in an additive graph, these differences were better appreciated (Supplementary Figure 1). While 50% of the aggregates for HY appear before fraction 7, DY aggregates do not do so until reaching fraction 9. Furthermore, whereas nearly 100% of aggregates from these two strains appear before fraction 12, less than 90% of them are present on that fraction for SSLOW prions. As expected, no PrP^27^,^28^,^29^,^30^ signals were observed when a brain homogenate from a healthy animal was submitted to this procedure (Fig. 1A, lower panel). In order to show that PK digestion was not affected by the presence of different concentrations of sucrose, we took complete, non-separated aliquots of HY prions and diluted them into fractions representing distinct sucrose concentrations. After treating each of these preparations with PK in the exact same conditions we observed that the resulting signals were similar in all cases, suggesting that PK activity was not affected by different sucrose concentrations (Supplementary Figure 2).

**Figure 2:**
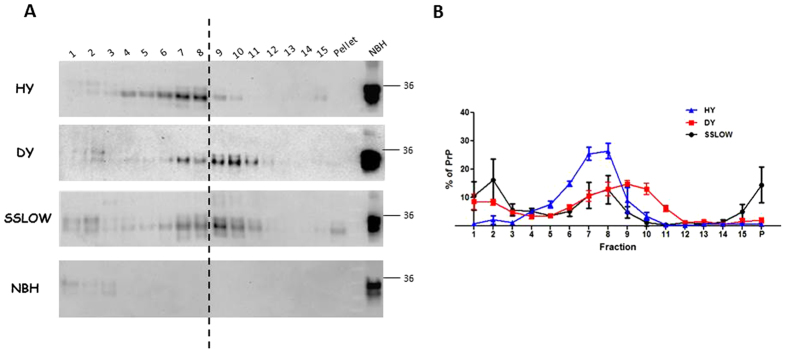
Sucrose-gradient fractionation of HY, DY and SSLOW prions in the absence of PK treatment. The same fractionated samples depicted in Fig 1 were analyzed for their PrP content in the absence of PK treatment. (**A**) Representative Western blots of the samples analyzed. The presence of PrP^C^ in fractions 1 and 2 is appreciated in all samples. Horizontal line at the right of the blot represents a 36 KDa molecular weight marker. Numbers at the top of the blots represent fraction numbers (from top to bottom). Vertical dotted lines represent blot splicing. (**B**) Fractionation profiles for samples corresponding to HY, DY and SSLOW prions developed without PK treatment. Data is represented as averages ± standard errors.

It has been shown that a potentially high proportion of PrP^Sc^ is sensitive to proteolytic degradation by PK^13^,^14^,^42^ and that the sensitivity/resistance to proteolysis may be different depending on the type of aggregates existing in the sample^12^,^30^,^31^. Since in the previous experiments we used PK treatment to detect the disease-associated form of PrP, it is possible that we might be underestimating the amount of PrP^Sc^ in some fractions. For this purpose, we analyzed the PrP content in each fraction by Western blotting without previous protease treatment. Importantly, the distribution profiles with or without PK treatment were similar for each strain (Fig. 2). The presence of PrP^C^ in each fractionation (including in the brain from a healthy animal) was clearly detected in the fractions containing the lowest concentration of sucrose (fractions 1 and 2). Interestingly, we observed that although not submitted to proteolysis, PrP signal in Western blot analyses of several fractions (3 through pellet) presented a lower molecular weight compared to PrP^C^. We believe that this effect is likely due to endogenous processing of misfolded PrP structures in brain tissue. When undigested whole brain extracts from prion infected animals are analyzed by Western blot, endogenous proteolytic processing is barely appreciated due to the presence of PrP^C^ in the sample. Importantly, our fractionation procedure was able to separate PrP^C^ from aggregated PrP, resulting in a more pronounced visualization of the effect mentioned above.

### Relationship between the aggregate distribution profiles and the biological properties of different prion strains

Incubation period to disease is one of the main features that differentiate prion strains^29^. Interestingly, we observed a direct correlation between the density of the most abundant species and the incubation period for each of these three prion strains (Table 1). Indeed, the highest proportion of aggregates in fractions of lower densities was related with shorter incubation periods and vice versa. Although our results do not account for the duration of the clinical stage, nor PrP^Sc^ brain tropism/toxicity, this data may suggest that the relative density of prion aggregates may be a factor regulating the time it takes for prions to produce the disease symptoms.

**Figure 3:**
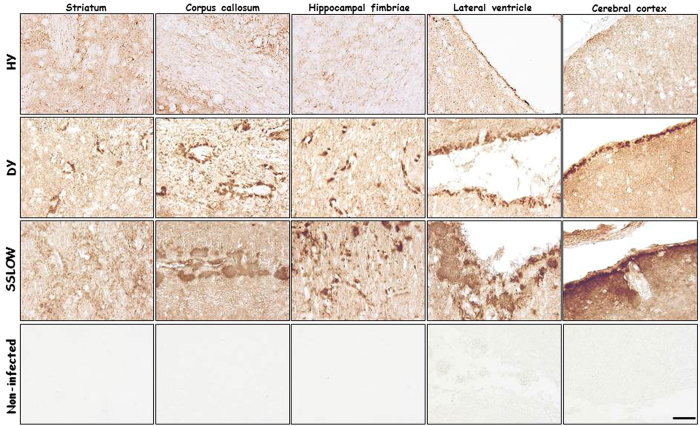
Strain specific PrP^res^ deposition in different brain areas. Representative pictures of PrP deposition in different brain regions of terminally ill animals infected with HY (n = 5), DY (n = 5), and SSLOW (n = 3) strains. Samples were processed with the 6H4 antibody after PK treatment. Brain sections from a non-infected animal (n = 3) are shown at the bottom panels as a control for staining-specificity against the PK-resistant form of the prion protein. The scale bar (50 μm) at the right-bottom panel is representative of all panels depicted in this figure.

The differential aggregate distribution found for these prion strains was also associated with the profile of PrP tissue deposition as observed by histological analyses. HY prions showed a mild synaptic and diffuse pattern of PrP^res^ deposition (Fig. 3, upper panels). The immunoreactivity found for HY-brains was more prominent in the middle layers of the frontal and occipital cortex, thalamus, caudate/putamen nuclei and granular layer of the cerebellum. Small plaque-like deposits were found in both corpus callosum and granular layer of the cerebellum. Also, some striatal neurons contained perinuclear plaque-like deposits of PrP in the perikaryon. Differentially, DY infected brains showed a more severe synaptic and diffuse pattern of PrP^res^ accumulation widespread throughout the brain (Fig. 3, second set of panels from top to bottom). Small, but abundant and more compact/amyloid-like plaques surrounded the lateral ventricle and the subpial region of the central nervous system, as well as the white matter of the cerebellum. Diffuse PrP immunostaining parallel to the axons of the granular neurons was observed in the molecular layer of the cerebellum. Finally, animals infected with SSLOW strain showed dense focal deposits of PrP in the striatum, hippocampal fimbriae and thalamus (Fig. 3, third set of panels from top to bottom). Consistent with previous studies^38^,^39^, a remarkable histopathological feature for SSLOW-brains was the presence of large/dense-core PrP plaques in the corpus callosum, around the lateral ventricle, and in the subependymal and subpial area of the brain, as well as in the periaqueductal subependymal region. Mild PrP^res^ deposition was found in the cerebellum. No PrP^res^ signal was found in the brain of non-infected animals (Fig. 3, bottom panels).

**Figure 4:**
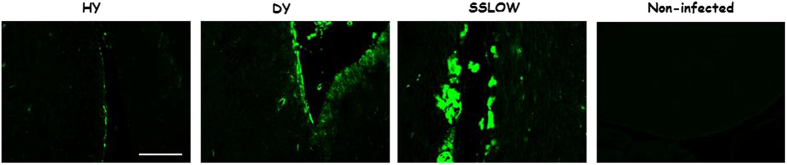
Amyloid deposition in brains of HY, DY and SSLOW infected animals. Representative pictures of HY (n = 5), DY (n = 5) and SSLOW (n = 3) brain sections depicting Thioflavin S positive PrP aggregates. Same treatment to brain slices of a non-infected animal is shown as negative control. All pictures shown correspond to the choroid plexus of the lateral ventricle. The scale bar (100 μm) at the right-bottom is representative for all panels.

In order to extend the immuno-histological observations, we stained HY, DY and SSLOW brain sections with thioflavin S (Fig. 4), a dye known to bind amyloid plaques with high specificity^43^. We observed that whereas HY brains showed few and small amyloid plaques (principally located in the molecular and granular layers of the cerebellar cortex), DY and SSLOW specimens showed the presence of larger and more abundant amyloid deposits highly positive for thioflavin S. DY brains showed perivacuolar and perivascular plaques in the striatum and thalamus, as well as some tracts of white matter, particularly in the corpus callosum and hippocampal fimbriae. As expected, the presence of large thioflavin S-reactive structures was found in the brain of SSLOW affected animals. No signal was obtained for brain tissues of an untreated (healthy) hamster. Although several features not explored in this study can account for the type of protein deposits accumulated in the brain of prion infected animals, our data suggests that the distinct population of PrP aggregates in terms of density found for different prion strains contributes to the histological pattern of PrP deposition generated in each case.

### Resistance to proteolysis is not linked to the presence of denser PrP aggregates

**Figure 5:**
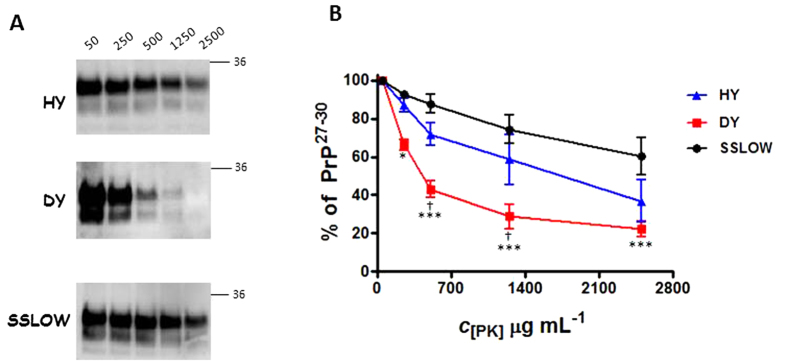
PK degradation profile of HY, DY and SSLOW prions. Extracts from HY, DY and SSLOW brains were treated with increasing concentrations of PK and remaining PrP^27^,^28^,^29^,^30^ was measured by analyzing the signal obtained after Western blotting. (**A**) Representative pictures of differential PK treatment for the three prion strains. Horizontal lines at the right of each blot indicate a 36 KDa molecular weight standard. Numbers at the top represent final PK concentrations in μg/mL. (**B**) PK resistance profile for HY, DY and SSLOW prions are represented as averages ± standard errors of three different measurements. No significant differences were found for HY and SSLOW prions at any PK concentration. (DY vs. HY prions: ^†^*p* < 0.05; DY vs. SSLOW prions: *p < 0.05, ***p < 0.001).

In order to correlate the aggregate distribution profile of PrP with biochemical properties of the misfolded protein, we measured and compared the relative resistance to PK degradation for these three different strains (Fig. 5). Our results indicated that HY prions have a higher resistance to degradation compared to the DY strain, in agreement with previous studies^44^,^45^. SSLOW prions also showed a strong resistance to PK treatment (approximately 70% of the signal remained intact after treating the sample with 2500 μg/mL of PK). These results do not reflect differences in the total amount of PrP present in the sample, since similar amounts by Western blot (signal at 50 μg/mL of PK) were loaded in the gel and all measurements were normalized compared to the lowest PK concentration. These results suggest that the proportion of differently packed PrP structures in a given strain does not correlate with its PK resistance.

### Analysis of the aggregation profile of HY prions at different clinical stages of the disease

**Figure 6:**
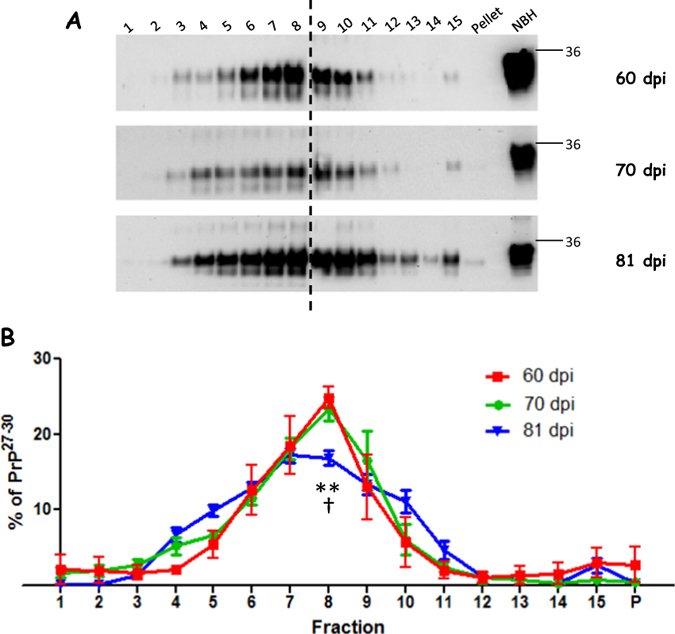
Aggregation profile of HY prions collected at different incubation periods. Brains from HY infected animals were collected at 60, 70 and 81 days after inoculation and distribution of PrP aggregates were analyzed by sucrose gradient fractionations. (**A**) Representative figures of PrP^27^,^28^,^29^,^30^ obtained after fractionating brain extracts from animals sacrificed at the denoted days post inoculation (dpi). Horizontal lines at the right of each blot represent a 36 KDa molecular weight marker. Numbers at the top of the blots represent fraction numbers (from top to bottom). Vertical dotted lines indicate blot splicing. The various gels were developed in the same membrane. (**B**) PrP^27^,^28^,^29^,^30^ aggregation profile for the three different groups analyzed. Results are represented as averages ± standard errors from either 4 (60dpi) or 5 (70 and 81dpi) animals per time group. 81 dpi results correspond to the same values depicted in Fig 1B for the HY strain. Values plotted were calculated from a single fractionation procedure per brain homogenate. No significant differences were found for 60 dpi and 70 dpi at any fraction. (60 dpi vs. 81 dpi: ^†^*p* < 0.05; 70 dpi vs 81 dpi: **p < 0.01).

It has long been established that formation of misfolded aggregates follows a seeding/nucleation mechanism in which formation of small seeding-competent oligomers is followed by a rapid growing of polymers to form large aggregates^46^,^47^. Extensive *in vitro* characterization has identified the formation of structures of increasing sizes when amyloid formation is assessed at different time points^28^,^48^. Extrapolating the *in vitro* results to the *in vivo* situation, a higher degree of packing and formation of denser structures are expected to appear over time. If this process is also occurring *in vivo*, we would expect that the distribution of aggregates should change with time in favor of denser aggregates at more advanced stages of the disease. Taking advantage of our sucrose gradient fractionation protocol, we wanted to characterize the dynamic distribution of prion aggregates *in vivo* at different stages of the disease. For that purpose, HY infected animals were sacrificed at different time points (60, 70 and 81 days) after infection. As expected, we observed that both clinical signs and amount of PrP^27^,^28^,^29^,^30^ in the brain of these animals increased with time (Supplementary Figure 3). However, the distribution of the PrP aggregates along the sucrose gradient showed little changes when analyzed at these different incubation periods (Fig. 6). No statistical differences in the PrP^27^,^28^,^29^,^30^ percentual content on any of the fractions analyzed was found for aggregates obtained at 60 or 70 days after HY prions administration. Descriptive and inferential statistics for both data sets confirmed that distributions were indistinguishable. However, fraction 8 for the 81 days post infection group showed significant differences with the other two sets of samples. These results indicate that HY prions partially maintain their strain-specific profile of aggregation throughout the course of the clinical stage, showing small changes only at advanced stages.

## Discussion

Although the identity of prions as proteinaceous particles is now widely accepted^38^,^49^,^50^,^51^, little information is available in regards of the specific structural arrangements that the infectious agent acquires. This lack of knowledge has limited our understanding of the molecular basis dictating prion strains. These prion variants are differentiated by their biochemical properties as well as the biological consequences they generate in the host^29^,^52^. Among them, we can include clinical symptoms, incubation periods, brain vacuolation profiles, and PrP^Sc^ resistance to denaturation and proteolysis, among others.

In this study we investigated whether three different hamster prion strains, containing PrP^Sc^ with the same amino acid sequence, could be differentiated by the profile of aggregates they accumulate in the brain. In this study we separated the aggregates by their density using equilibrium sedimentation in sucrose gradients. Density of protein aggregates is a consequence of several features, including their size, degree of packing and hydration. These features can be modulated by the proportion of β-sheets contained in each structure. Differently from previous reports, we avoided extensive pre-manipulation of the brain extracts, such as treatment with PK, sonication, reducing agents, and/or centrifugation^11^,^12^. Several reports have shown that such procedures on PrP^Sc^ do not affect their strain specific properties when infecting animals^53^,^54^. However, sonication of PrP^Sc^ may change incubation periods likely by modifying the size distribution of the infectious protein aggregates^55^. The goal of this study was to obtain a closer picture of the particles present in the brain of diseased animals by performing minimal manipulation on brain extracts. We found that the three different hamster strains included in this study had distinguishable PrP-aggregates distribution profiles. In all strains, PrP^Sc^ aggregates distributed in a Gaussian-like fashion, indicating that they correspond to a continuum of polymers (Fig. 1). HY material peaked in fraction 7–8. SSLOW behaved in a similar way (peaking in fraction 8) but having a sizeable amount of aggregates in the pellet fraction. DY prions peaked at fraction 9 in a distinguishable pattern compared to the other prion agents. These results suggest that conformational differences on these aggregates lead to a differential distribution profile of aggregates in terms of density. Although our data strongly point to that direction, we cannot rule out that each PrP type possesses a particular propensity to bind other molecules (i.e. lipids) that could be responsible for sedimentation differences. In our study we attempted to eliminate this variable with a mild detergent treatment in addition to removing tissue debris.

Previous reports have shown that ultracentrifugation in sucrose density gradients at extended times can separate protein aggregates mostly by their mass^56^. Our results are in agreement with mass separation since PrP^C^, a molecule expected to exist mostly as a monomer, was found only in fractions containing the lowest concentration of sucrose (Fig. 2). Nevertheless, it is important to note that molecular weights cannot be directly extrapolated from sucrose gradient fractionation results because other variables besides the size (i.e. hydration and packing) determine the density fraction in which the material appear. Regardless, we observed that biochemical assessment of the aggregates by the sucrose density gradient fractionation method correlated well with both the histological analysis of protein deposits and incubation periods. Large amyloid plaques observed in the brains of SSLOW infected animals were not found in the HY brains, with DY materials exhibiting an intermediate behavior. Although the presence of plaque-like deposits in SSLOW was not widespread, it was identified in several anatomical structures. It is likely that the material present in the pellet of SSLOW correspond to the dense amyloid plaques observed by histology. The extensive diffuse synaptic staining typically observed in the HY strain probably corresponds to the structures appearing in the middle of the gradient (fractions 7 and 8). Due to the minimal manipulation performed in the samples before fractionation, we suggest that our procedure provides a faithful picture of the whole population of aggregates and deposits present in the brain of prion infected animals.

Interestingly, our results also showed a positive correlation between the density of aggregates and the incubation period to established clinical signs of the disease. One interpretation of these results is that smaller, less compacted aggregates are better seeds that more efficiently self-propagate to induce pathology. An alternative possibility is that longer incubation periods in SSLOW compared to DY and especially HY allowed this strain to continue to later stages of the aggregation process resulting in denser and more packed deposits. While these correlations look attractive, our measurements involve only three prion strains and do not consider the specific tropism and intrinsic toxicity of the aggregates associated with each particular infectious agent. Further studies using additional prion strains from other prion susceptible animals will corroborate/discard whether this behavior is common for all prion variants.

An important question regarding the distribution of aggregates is which structures are the most efficient in sustaining prion replication. Several *in vitro* and *in vivo* experiments have shown that protein oligomers are the most neurotoxic structures and also perhaps the best nuclei for aggregation^20^,^57^,^58^. In this line, it has been reported that certain prion strains exhibiting high oligomeric content produce shorter incubation periods^12^,^30^,^31^. Nevertheless, little is known about the contribution of different types of aggregates to prion replication and infectivity. A previous report by Laferriere *et al.* showed that ovine prion strains generated in transgenic mice overexpressing ovine PrP^C^ and causing clinical disease at different times after inoculation were indistinguishable in terms of the density profiles of their PrP aggregates^15^. On the contrary, our results studying HY, DY and SSLOW hamster prion strains showed a clear correlation between the profile of aggregates and incubation periods (Table 1). As previously discussed, longer incubation periods could allow prion aggregates to generate highly packed structures as judged by our fractionation and histology data. In that line, it has been shown that mice overexpressing PrP from other species produces different profiles of PrP deposition when compared to the ones generated by the naïve species^59^,^60^. The fast generation of infectivity in PrP overexpressing mice could explain why no differences in the density of aggregates were described in the previously mentioned report^15^. Further studies using a larger number of clinically diverse prion strains generated in naïve hosts should be useful to confirm our results.

An important biochemical and pathogenic feature associated with prions involve its partial resistance to proteolytic degradation. It is well-established that prion strains have a differential sensitivity to proteolysis^29^,^44^,^45^. The leading explanation for this behavior is that PK resistance is dependent on the size of aggregates produced, with large fibrillar structures being more resistant to degradation than small oligomers. An alternative explanation for differential proteolytic resistance might be that differences reside on the specific conformation and degree of packing that each strain acquires. Our results indicate that a simple relationship between the distribution of aggregates for a given prion strain and PK resistance cannot be made. Thus, our data indicate that PK resistance is independent of the size/density of aggregates and differences possibly lie in the specific PrP^Sc^ conformation.

An interesting and unexpected result from our studies was that the distribution of PrP species does not appear to change substantially in distinct stages of the disease. Since the formation of aggregates is expected to increase with the progressive accumulation of proteins we hypothesized that a pronounced shift towards less dense aggregates will occur at earlier stages of the disease. However, our results showed that the PrP distribution in sucrose gradients for animals sacrificed at different time points after HY prion infection (from the pre-symptomatic to the severe stage of the disease) was similar in two out of the three time points analyzed (Fig. 6). The only change was found for the longest incubation period where significant differences were found in the fraction peaking PrP^27^,^28^,^29^,^30^ content (fraction 8). These findings indicate that, once formed, the distribution of PrP^Sc^ species is maintained over time and is minimally affected by the progressive accumulation of the agent. The interpretation of this data is that the packaging associated with distinct strains depends mostly on the specific structural information encoded in the PrP^Sc^ molecule. The data may also suggest that some of the species present in low concentration sucrose fractions might be off-pathway, stable structures that are not precursors for denser aggregates. The shorter incubation periods of HY prions compared to the other strains analyzed may also contribute to the minimal changes observed in the PrP distribution over time.

The data presented here confirms that prion strains generate different types of aggregates that can be differentiated by their intrinsic density. An orchestrated balance among several properties, including density of PrP aggregates, brain tropism, toxicity, resistance to degradation, and seeding efficiency may play a role in the defined strain-unique features generated by a particular prion agent. The study of the dynamic distribution of aggregates and their associated biological and biochemical properties may serve not only to increase our knowledge of this intriguing infectious agent, but also help in the development of diagnostic and therapeutic methods directed to specifically target key structural properties of prions.

## Additional Information

**How to cite this article**: Morales, R. *et al.* Strain-dependent profile of misfolded prion protein aggregates. *Sci. Rep.* **6**, 20526; doi: 10.1038/srep20526 (2016).

## Supplementary Material

Supplementary Information

## Figures and Tables

**Table 1:** Sucrose-gradient distribution and incubation periods of HY, DY, and SSLOW prions in Syrian hamsters.

Strain	Fractions 4–5 (% PrP^27–30^)	Fractions 10–11 (% PrP^27–30^)	Pellet (% PrP^27–30^)	Incubation period (days)*
HY	16.6 ± 1.5	15.9 ± 2.2	0.4 ± 0.1	73,8 ± 1,6
DY	3.7 ± 0.6	33.6 ± 4.0	0.1 ± 0.1	228,6 ± 3,7
SSLOW	8.7 ± 0.2	11.1 ± 2.1	11.9 ± 4.5	432,7 ± 9,3

Values are expressed as means ± standard errors after fractionating 5 (HY, DY) or 3 (SSLOW) different hamster brains.

^*^Incubation periods represent a subsequent infectivity passage of the samples used for size-fractionation (n = 5/group). Data is expressed as means ± standard errors of the time when animals were sacrificed as explained in Materials and Methods.
