# Supplementary material for: Strain-dependent profile of misfolded prion protein aggregates
Source: Sci Rep. 2016 Feb 15;6:20526. doi: 10.1038/srep20526 (PMC4753423; doi:10.1038/srep20526)

SUPPLEMENTARY INFORMATION

Strain-dependent profile of misfolded prion protein aggregates

Rodrigo Morales[+,*], Ping Ping Hu[+], Claudia Duran-Aniotz, Fabio Moda, Rodrigo Diaz-Espinoza, Baian Chen, Javiera Bravo-Alegria, Natallia Makarava, Ilia V. Baskakov and Claudio Soto[*].

+These authors contributed equally to this work.

* Address correspondence to: Rodrigo Morales, Rodrigo.MoralesLoyola@uth.tmc.edu; or Claudio Soto, Claudio.Soto@uth.tmc.edu.

SUPPLEMENTARY FIGURE LEGENDS

Supplementary Figure 1. Cumulative distribution of $PrP^{27\text{-}30}$ aggregates for HY, DY and SSLOW prions. Data depicted in Supplementary Table 1 was plotted as accumulative values along the gradient. Dashed and dotted lines represents 50% and 90% of total aggregation, respectively.

Supplementary Figure 2. PK activity is not affected by different sucrose concentrations. Sucrose fractionation procedure was performed using a non-infected brain homogenate in replacement of diseased samples and fractions were collected. Concentrated HY preparations were spiked in fractions 3, 5, 8 and 14 and submitted to PK treatment (final PK concentration: 50 µg/mL). **A**, Representative figure showing a set of PK treated samples in the presence of different sucrose fractions. Horizontal line at the right of the blot represents a 36 KDa molecular weight marker. **B**, Densitometric analysis of the signal obtained after 4 independent assays. 100% was assigned to the PrP signal obtained after digesting the same sample in PBS.

Supplementary Figure 3. Clinical evolution and $PrP^{27\text{-}30}$ accumulation in HY-infected animals sacrificed at different time points. HY-infected hamsters were sacrificed at different times after infection. Clinical signs and $PrP^{27\text{-}30}$ content in their brains were measured. **A**, Clinical evolution of hamsters sacrificed at different time points after infection. 5 different animals were included in each time point and the values correspond to the clinical scale used to measure the behavioral abnormalities from normal animal (1) to severe stage of the disease (4), as described in Materials and Methods. Data is expressed as averages ± standard errors. **B**, Presence of $PrP^{27\text{-}30}$ in brains of animals sacrificed at different time points. Arrows at the right of the blot represent molecular weight markers. Each lane in each blot represents a different animal.

Supplementary Table 1. Sucrose-gradient distribution of HY, DY, and SSLOW prions in Syrian hamsters.

	Hyper	Drowsy	SSLOW
Fraction 1	0.15 ± 0.1	0.74 ± 0.3	0.14 ± 0.1
Fraction 2	0.20 ± 0.1	0.76 ± 0.4	0.18 ± 0.1
Fraction 3	1.35 ± 0.3	0.91 ± 0.3	1.10 ± 0.7
Fraction 4	6.75 ± 1.0	1.48 ± 0.4	4.24 ± 0.6
Fraction 5	9.89 ± 0.8	2.21 ± 0.3	4.44 ± 0.5
Fraction 6	12.95 ± 0.7	4.34 ± 0.7	9.37 ± 2.1
Fraction 7	17.24 ± 1.2	10.70 ± 1.8	16.68 ± 2.5
Fraction 8	16.90 ± 1.3	15.02 ± 2.0	22.19 ± 1.6
Fraction 9	13.44 ± 1.4	21.16 ± 2.0	14.77 ± 1.4
Fraction 10	11.18 ± 1.5	19.03 ± 2.4	7.79 ± 1.5
Fraction 11	4.73 ± 1.2	14.53 ± 1.9	3.33 ± 0.6
Fraction 12	1.18 ± 0.4	3.18 ± 1.0	0.87 ± 0.1
Fraction 13	0.72 ± 0.4	2.03 ± 0.4	0.15 ± 0.04
Fraction 14	0.33 ± 0.1	0.77 ± 0.3	0.13 ± 0.1
Fraction 15	2.66 ± 1.0	2.51 ± 0.6	2.71 ± 1.3
Pellet	0.36 ± 0.1	0.09 ± 0.1	11.93 ± 4.5

Data for each fraction is represented as the percentage of PrP^{27-30} signal obtained as explained in Materials and Methods. Values are expressed as means ± standard errors after fractionating 5 (HY, DY) or 3 (SSLOW) different hamster brains.

Supplementary Table 2. Statistical analyses on sucrose-gradient distribution for HY, DY, and SSLOW prions in Syrian hamsters.

	Hyper vs. Drowsy	Hyper vs. SSLOW	Drowsy vs. SSLOW
Fraction 1	ns	ns	ns
Fraction 2	ns	ns	ns
Fraction 3	ns	ns	ns
Fraction 4	*	ns	ns
Fraction 5	***	*	ns
Fraction 6	***	ns	ns
Fraction 7	***	ns	*
Fraction 8	ns	*	**
Fraction 9	***	ns	**
Fraction 10	***	ns	***
Fraction 11	***	ns	***
Fraction 12	ns	ns	ns
Fraction 13	ns	ns	ns
Fraction 14	ns	ns	ns
Fraction 15	ns	ns	ns
Pellet	ns	***	***

Data was analyzed by Two-Way ANOVA, followed by Bonferroni post-test. * P<0.05; ** P<0.01; *** P<0.001; ns: no-significance.

Supplementary Figure 1

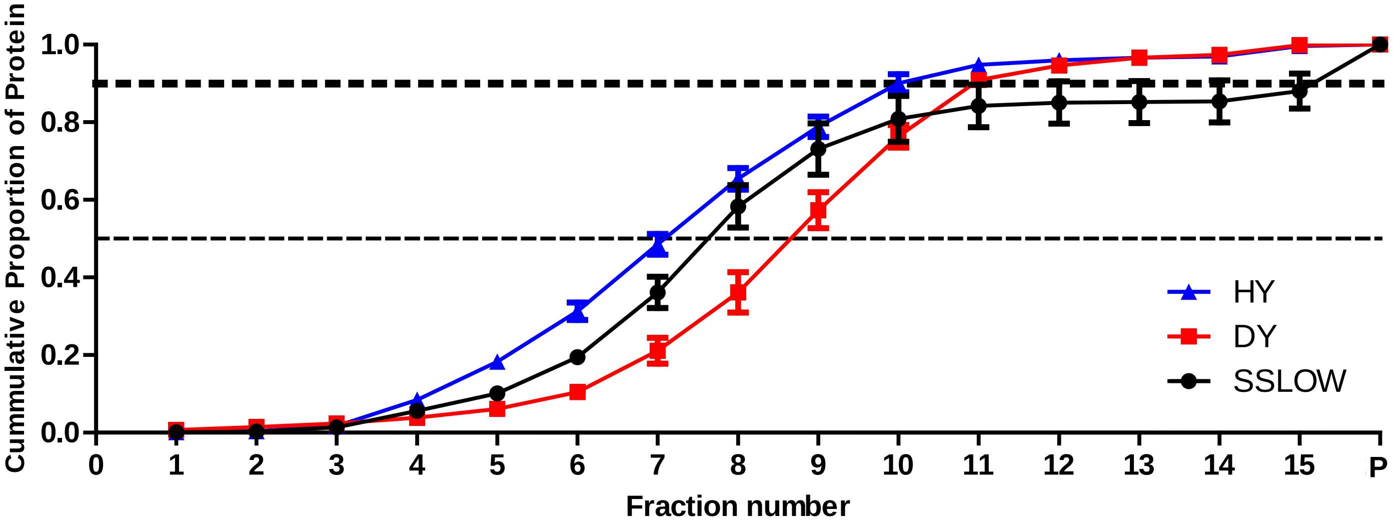

Supplementary Figure 2

A

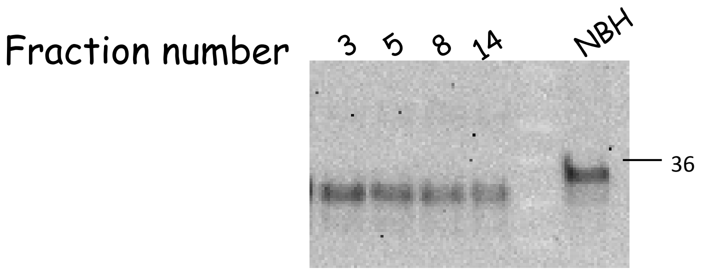

B

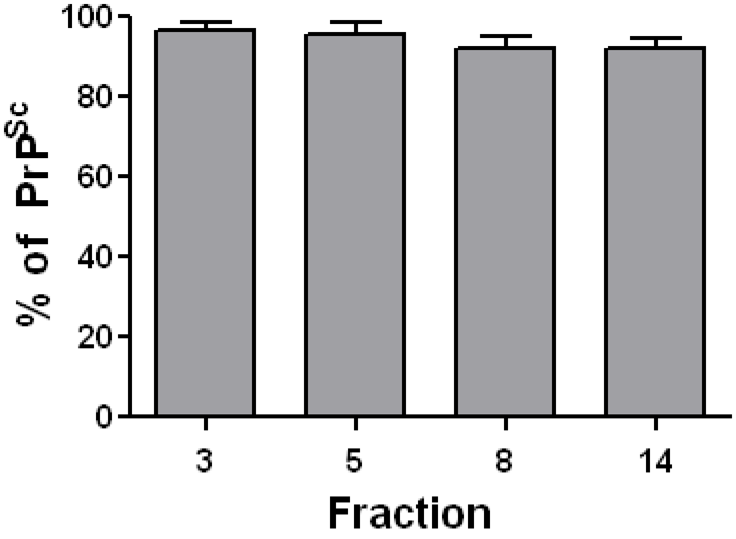

Supplementary Figure 3

A

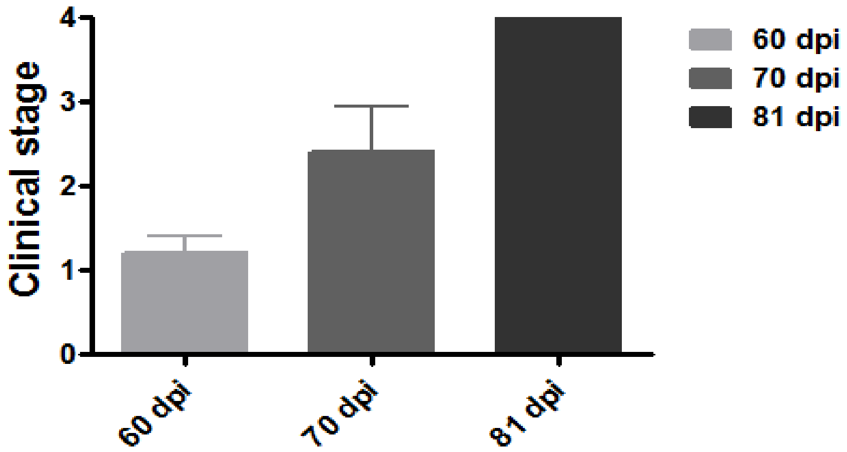

B

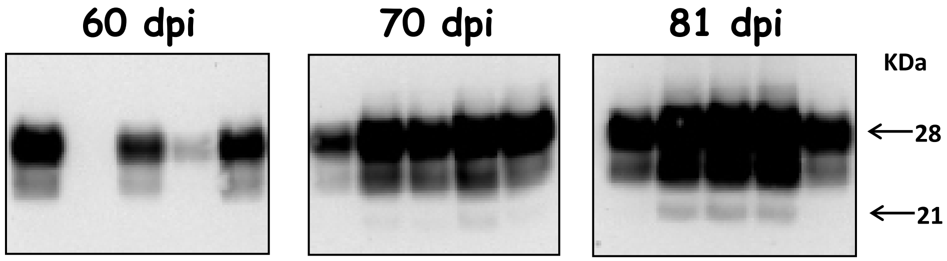

Supplement: Supplementary Information [file srep20526-s1.pdf]
